# Profiling Genome-Wide Methylation Patterns in Cattle Infected with *Ostertagia ostertagi*

**DOI:** 10.3390/ijms26010089

**Published:** 2024-12-26

**Authors:** Clarissa Boschiero, Ethiopia Beshah, Xiaoping Zhu, Wenbin Tuo, George E. Liu

**Affiliations:** 1 Animal Parasitic Diseases Laboratory, Beltsville Agricultural Research Center, Agricultural Research Service, United States Department of Agriculture, Beltsville, MD 20705, USA; 2 Department of Veterinary Medicine, University of Maryland, College Park, MD 20742, USA; 3 Animal Genomics and Improvement Laboratory, Beltsville Agricultural Research Center, Agricultural Research Service, United States Department of Agriculture, Beltsville, MD 20705, USA

**Keywords:** cattle, *O. ostertagi*, immune response, DNA methylation, duodenum, lymph nodes, abomasum

## Abstract

DNA methylation (DNAm) regulates gene expression and genomic imprinting. This study aimed to investigate the effect of gastrointestinal (GI) nematode infection on host DNAm. Helminth-free Holstein steers were either infected with *Ostertagia ostertagi* (the brown stomach worm) or given tap water only as a control. Animals were euthanized 30 days post-infection, and tissues were collected at necropsy. We conducted epigenome-wide profiling using a mammalian methylation array to explore the impact of infection on methylation patterns in the mucosa from abomasal fundus (FUN), pylorus (PYL), draining lymph nodes (dLNs), and the duodenum (DUO). The analysis covered 31,107 cattle CpGs of 5082 genes and revealed infection-driven, tissue-specific, differential methylation patterns. A total of 389 shared and 2770 tissue-specific, differentially methylated positions (DMPs) were identified in dLN and FUN, particularly in genes associated with immune responses. The shared DMPs were found in 263 genes, many of which are involved in immune responses. Furthermore, 282, 244, 52, and 24 differentially methylated regions (DMRs) were observed in dLN, FUN, PYL, and DUO, respectively. More hypomethylated DMRs were detected in dLN and FUN, while more hypermethylated DMRs were found in PYL and DUO. Genes carrying DMPs and DMRs and enriched pathways relating to immune functions/responses were detected in infected animals, indicating a link between DNA methylation and the infection. The data may implicate a crucial role of DNAm in regulating the nature/strength of host immunity to infection and contribute to a deeper understanding of the epigenetic regulatory landscape in cattle infected by GI nematodes.

## 1. Introduction

Epigenetic modifications can alter gene expression without changing the underlying DNA sequence, exerting a profound impact on various biological processes, including development, aging, and disease. Common epigenetic modifications include DNA methylation (DNAm), histone modifications, and non-coding RNA regulation [1]. DNAm emerges as a prominently investigated mechanism, serving a crucial role in upholding genome stability, silencing transposable elements, initiating X-chromosome inactivation, and ensuring normal growth and development [2,3]. DNAm patterns are not permanent and can change throughout the life of an individual, which can be a response to environmental modifications or associated with aging or disease [3]. These modifications occur at promoters, transposons, enhancers, and silencers with CpG dinucleotides being the primary site for DNAm in mammals [2]. Approximately 40% of the mammalian genes contain CpG islands and clusters of CpG sequences in their promoters and exonic regions [3,4].

A diverse range of technologies, such as methylation-specific PCR (MSP), methylated DNA immunoprecipitation (MeDIP), bisulfite sequencing, and methylation arrays, has been utilized in genome-wide research studies for the analysis of DNA methylation patterns [5]. Bisulfite sequencing is considered a gold standard for DNAm detection but has limitations including sensitivity to degradation of DNA samples and high costs [5]. However, methylation arrays are cost-efficient with reliable and reproducible results [5,6,7,8,9,10].

The mammalian methylation array enables the interrogation of CpG sites at a single-nucleotide resolution, offering robust measurements of DNA methylation in over 200 species, including cattle. This array provides deep coverage of conserved cytosines and focuses on sequences with a high degree of conservation [6]. The CpG probes selected in the mammalian methylation array were specifically chosen, focusing on sequences with a high degree of conservation [6]. The gene set included in this array is associated with pivotal biological processes, including development, growth, transcriptional regulation, metabolism, cancer, mortality, and aging [6]. It has proven effectiveness in constructing epigenetic clocks in various species [7,8,9,11] and in investigating DNAm patterns in cattle [10].

In cattle, DNAm studies have explored associations with diseases [12,13,14], aging [15], reproduction [16,17,18], embryonic development [19,20], muscle development [21], and responses to heat stress [22]. Infections by pathogens, including parasites, are able to influence the host-cell transcriptome and epigenome by modulating host immune responses [23]. In a recent investigation, the influence of cattle parasites on DNAm was assessed, revealing a potential correlation between host resistance to parasite infection and global DNAm [24]. However, the relative levels of methylated DNA were detected using an antibody-based method, which only detects large changes in global DNAm [5].

This study aims to characterize DNAm patterns in four different tissues of cattle infected with the stomach nematode parasite, *Ostertagia ostertagi*, using a custom-designed mammalian methylation array that identifies differentially methylated regions potentially responding to the infection and regulating the infection-associated, tissue-specific gene expression.

## 2. Results

### 2.1. Infected Animals and Parasitology

The trickle infection with *O. ostertagi* L3 larvae for 4 weeks resulted in an average fecal egg count of 53.5 ± 5.6 eggs per gram (EPG) between 22 and 30 dpi and a mean worm load of 2496 ± 693 per animal at 30 dpi when the animals were euthanized. The infection caused a reduction in the daily body weight gain (infected group: 0.98 ± 0.10 kg/day; control group: 0.52 ± 0.10 kg/day). At necropsy, animals in the infected group had a significant increase in abomasal content pH (infected group: 4.6 ± 0.1; control group: 3.4 ± 0.5) and total dLN weight (infected group: 69.7 ± 7.1 g; control group: 5.6 ± 1.0 g).

### 2.2. Cattle CpG Probes

**Figure 1:**
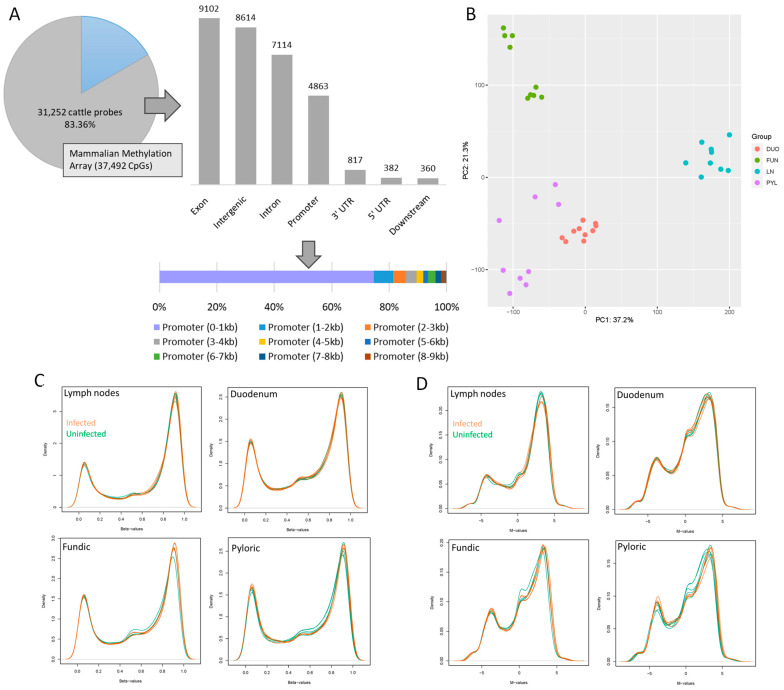
Overview of the cattle CpGs probes mapped to the cattle genome. (**A**) Number of cattle probes from the mammalian methylation array, along with respective genome annotation. (**B**) PCA of the 37 samples analyzed in this study based on the β-values. (**C**) The β-value distribution of 37 individual samples from infected and uninfected groups. (**D**) The M-value distribution of 37 samples from infected and uninfected animals, where infected samples are depicted in green, and uninfected samples are shown in orange.

In this study, the custom Infinium array “HorvathMammalMethyl-Chip40” (mammalian methylation array) [6] was utilized to obtain DNA methylation data from four tissues (dLN, DUO, FUN, and PYL) of cattle infected with *O. ostertagi* and uninfected control animals. From the original 37,492 CpGs present across over 200 species, a total of 31,252 CpGs was mapped to the cattle genome (ARS-UCD1.2) [25], representing ~83% of the probes (Figure 1A). Six probes were removed due to their locations on the unplaced sequence, resulting in 31,246 CpGs associated with 5089 genes. The annotation of CpG probes revealed that a majority of cattle probes were situated within exons (29.12%), intergenic regions (27.56%), and introns (22.76%) (Figure 1A). Probes located in promoter regions (15.56%) predominantly occurred within 1 kb of TSS (11.60%). Then, the 31,246 CpGs were normalized with the SeSaMe package (version 1.15.1) [26], generating the β-value of each probe for the methylation levels. Quality control was performed on all samples and the resultant 31,246 CpGs. The PCA conducted on the β-values of the 37 samples showed distinct clusters corresponding to the different tissues (Figure 1B). The first PC explained 37.2% of the variance, while the second PC explained 21.3% of the variance, effectively separating samples according to tissue types. The distribution analysis of the β-values and M-values for each tissue and sample displayed three peaks, indicating similar distribution patterns of β- and M-values between infected and uninfected groups (Figure 1C,D).

### 2.3. Probe Filtration at SNP Sites

Two different steps were utilized to remove the impact of SNPs on CpG probes. First, we compared the 31,246 cattle CpGs against the location of 67,965,046 SNPs identified in 1168 Holsteins with MAF > 5%. We found that 103 probes contain SNPs. Subsequently, using the MethylToSNP package (version 0.99.0) [27], we identified an additional 36 probes that are potentially affected by SNPs. Consequently, we removed a total of 139 CpGs that are confounded by adjacent SNPs, leaving a total of 31,107 cattle CpGs that correspond to a total of 5082 cattle genes for the subsequent analysis.

### 2.4. DMPs Specific to Infected Animals

**Figure 2:**
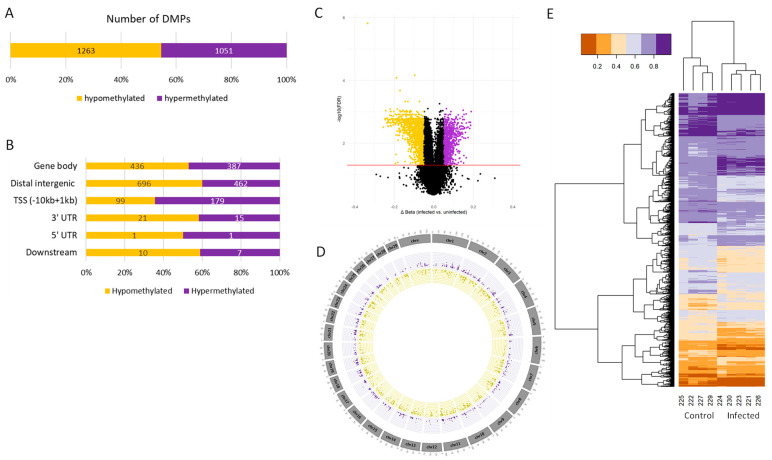
Overview of the dLN DMPs showing the counts of hypomethylated (yellow) and hypermethylated (purple) DMPs. (**A**) Number of hypomethylated and hypermethylated DMPs (FDR < 0.05 and |Δβ| > 5%). (**B**) DMP annotation (−10 kb to +1 kb from the nearest TSS) for both hypomethylated and hypermethylated DMPs. (**C**) Volcano plots of the DMPs, where black dots represent the DMPs that are not significantly differentially methylated, while the yellow or purple dots indicate the DMPs that are significantly hypermethylated or hypomethylated (FDR < 0.05 and |Δβ| ≥ 5%). The red line represents FDR < 0.05. (**D**) Circular plots of the genome distribution of the hypomethylated and hypermethylated DMPs. (**E**) Heatmaps of the hypomethylated and hypermethylated DMPs for each animal in either the control or the infected group. The legend indicates the beta coefficient values.

**Figure 3:**
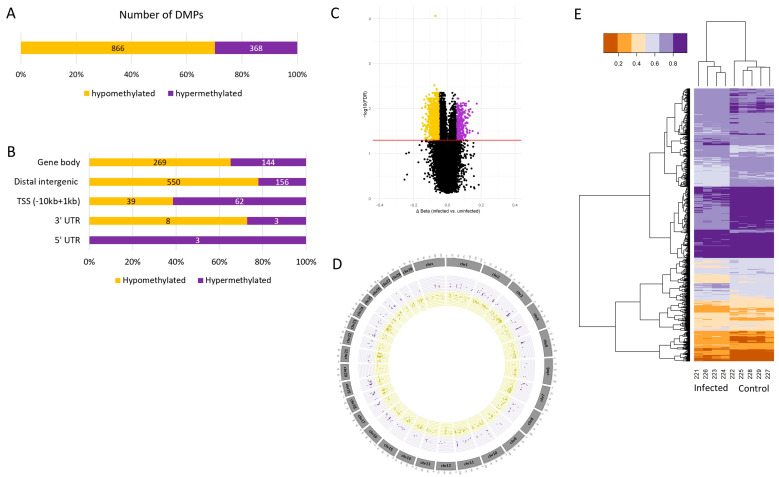
Overview of the FUN DMPs showing the counts of hypomethylated (yellow) and hypermethylated (purple) DMPs. (**A**) Number of hypomethylated and hypermethylated DMPs (FDR < 0.05 and |Δβ| > 5%). (**B**) DMP annotation (−10 kb to +1 kb from the nearest TSS) for both the hypomethylated and hypermethylated DMPs. (**C**) Volcano plots of the DMPs where black dots represent the DMPs that are not significantly differentially methylated, while the yellow or purple dots indicate the DMPs that are significantly hypermethylated or hypomethylated (FDR < 0.05 and |Δβ| ≥ 5%). The red line represents FDR < 0.05. (**D**) Circular plots of the genome distribution of the hypomethylated and hypermethylated DMPs. (**E**) Heatmaps of the hypomethylated and hypermethylated DMPs for each animal in either a control or an infected group. The legend indicates the beta coefficient values.

A total of 2314 infection-specific DMPs (FDR < 0.05) were identified in dLN, which include 1051 hypermethylated (Δβ ≥ 5%) and 1263 hypomethylated (Δβ ≤ −5%) sites (Figure 2A). For the FUN, 1234 infection-specific DMPs (FDR < 0.05) were detected, including 866 hypermethylated and 368 hypomethylated sites (Figure 3A). No DMPs were observed in the PYL and DUO tissues. Among the detected DMPs, 389 DMPs located in 263 genes (12.3%) were shared between dLN and FUN tissues (Table S1).

The annotation of these DMPs within a range of −10 kb to +1 kb from the nearest TSS revealed that the 1234 FUN DMPs are associated with 674 genes, and the 2314 dLN DMPs are associated with 1041 genes (Table S1). The DMP annotation showed that the majority of the hypermethylated DMPs were located in intergenic regions and gene bodies (intron) for both tissues (Figure 2B and Figure 3B).

We further investigated the DMPs that were entirely located on genes or TSS (−10 kb to +1 kb from the TSS). Among the FUN DMPs, 276 were entirely located on 177 genes, with 101 situated on the TSS. In dLN, 609 DMPs were entirely located on 321 genes, and 278 were on the TSS (Table S2).

The circular plots depicting the genome distribution of hypermethylated and hypomethylated sites exhibit a balanced distribution in the FUN tissue and dLN (Figure 2D and Figure 3D). However, a higher concentration of DMPs was observed on chromosomes 2, 3, 4, 11, and 18 for FUN and chromosomes 1, 2, 3, 7, and 11 for dLN. The heatmaps from both tissues show two distinct clusters of animals based on their beta coefficient values, segregating the infected and uninfected groups (Figure 2E and Figure 3E).

Among the 2314 infection-specific DMP genes identified in the dLN, several known immune-related genes were present, including *C8B*, *IFNG*, *IGBP1*, *IL20RB*, *INHBA*, *IRF1*, *IRF5*, *LEF1*, *LOC534155*, *LPXN*, *NCF2*, *RBPJL*, *TAGAP*, *TLX3*, and *XCL1* (Table S1). The top five genes with hypermethylated CpGs in dLN were cg07975023 (*RAPGEF2*), cg13702222 (*MBNL1*), cg03742417 (*MBNL1*), cg18441511 (*SATB1*), and cg22199101 (*FKBP5*). In the FUN, among the 1234 DMPs, several immune-related genes were found, including *ANXA1*, *ANXA3*, *C8B*, *ICOS*, *IFNG*, *IFNGR1*, *IGSF1*, *IL17B*, *IL20RB*, *INHBA*, *TNFAIP6*, *TNFSF8*, and *TXNL1* (Table S1). The top five genes with hypermethylated CpGs in the FUN were cg11730351 (*CRTAC1*), cg05821475 (*MIR2400*), cg05562961 (*RNF220*), cg05107826 (*SMAD6*), and cg26808264 (*DAPL1*).

To understand the biological functions of the DMPs, all genes annotated by DMPs were analyzed for overrepresentation using PANTHER (FDR < 0.05) and IPA. A total of 674 genes from the 1234 FUN DMPs and 1041 genes from the 2314 dLN DMPs were examined. The overrepresentation analysis displayed similar results for both tissues, with 12 enriched GO families for MF, particularly for DNA binding and transcription regulations (Table S3).

The IPA analysis generated 21 significant networks (score > 10) for dLN and 20 networks for FUN (Table S4). Networks in the dLN were related to the cell cycle, cell morphology, cell death, cancer, cellular development, and humoral immune response. Networks in the FUN were related to cancer, cellular development, cell signaling, cell death, and immunological and gastrointestinal (GI) diseases.

Furthermore, IPA revealed enriched canonical pathways in both dLN and FUN, including several immune-related pathways (Table S5). In the FUN, 53 enriched canonical pathways were identified (*p*-value < 0.01), including pathways of WNT/β-catenin signaling, CDX GI cancer signaling, chronic myeloid leukemia signaling, and polyamine regulation in colon cancer. In dLN, 115 enriched canonical pathways were found (*p*-value < 0.01), including pathways like WNT/β-catenin signaling, chronic myeloid leukemia signaling, the CDX gastrointestinal cancer signaling pathway, IL-12 signaling and production in macrophages, Th1 and Th2 activation, IL-7 signaling, and IL-4 and IL-13 signaling.

### 2.5. Infection-Specific DMRs

**Figure 4:**
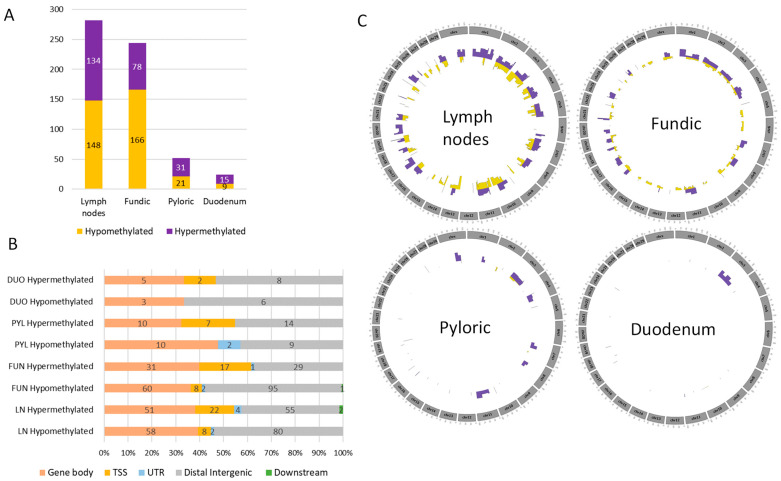
Overview of the fundic DMRs showing the hypomethylated (yellow) and hypermethylated (purple) regions. (**A**) Number of hypomethylated and hypermethylated DMRs (*p*-value ≤ 0.001 and |Δβ| ≤ 10%) identified across four cattle tissues. (**B**) DMR annotation (−10 kb to +1 kb from the nearest TSS) showing the hypomethylated and hypermethylated DMRs in each tissue (LN: lymph nodes; FUN: fundic; PYL: pyloric; DUO: duodenum). (**C**) Circular genome distribution plots of the hypomethylated and hypermethylated DMRs in each tissue.

Then, we identified infection-specific DMRs (*p*-value ≤ 0.001) with a methylation cut-off of 0.1, corresponding to a 10% difference in the methylation levels. dLN and FUN tissues showed more identified DMRs compared to PYL and DUO tissues (Figure 4A). Specifically, in dLN and FUN tissues, more hypomethylated DMRs (Δβ ≤ −10%) were found, totaling 148 and 166, respectively (Figure 4A). On the other hand, in the PYL and DUO, more hypermethylated DMRs (Δβ ≥ 10%) were identified, totaling 31 and 15, respectively (Figure 4A).

Annotation of these DMRs within the −10 kb to +1 kb from the nearest TSS revealed their association with genes: 282 dLN DMRs are involved with 214 genes, 244 FUN DMRs are involved with 206 genes, 52 PYL DMRs are involved with 48 genes, and 24 DUO DMRs are involved with 24 genes (Table S6). The annotation showed that the majority of the hypermethylated DMPs were located in intergenic regions, except for FUN, where most hypermethylated DMRs were on gene bodies (Figure 4B).

We further investigated genes that either themselves or whose TSS regions (−10 kb to +1 kb from the TSS) entirely contained the DMRs. We classified them as either differentially methylated genes (DMGs) or differentially promoter-methylated genes (DPMGs), respectively. We identified 83 DMGs (in 67 unique genes) and 30 DPMGs in dLN; 55 DMGs (in 51 genes) and 25 DPMGs in the FUN; 16 DMGs (in 15 unique genes) and 7 DPMGs in PYL; and 4 DMGs and 2 DPMGs in DUO (Table S7).

The circular genome distribution plots of the hypermethylated and hypomethylated regions show an even distribution in the FUN and dLN (Figure 4C). However, more DMRs were located on chromosomes 1, 2, 3, 7, and 11 for FUN and chromosomes 3, 4, 8, 11, and 21 for dLN.

Among the 214 dLN-specific DMR genes identified in the infected cattle, immune-related genes such as *BCL11A*, *FKBP5*, *FOXP1*, *IGBP1*, *IRX5*, *OIT3*, *RAB2A*, *RARA*, *SMAD2/4*, *TLE1/4*, and *ZEB2* were notable (Table S6). The top five genes with hypermethylated DMRs in dLN included *RARA*, *SMAD2*, *RPS3A*, *DYRK1A*, and *MBNL1*. In the 206 FUN-specific DMR genes from infected cattle, immune-related genes included *C8B*, *EGR3*, *FKBP5*, *FOSB*, *FOXP1*, *IL17B*, *IL1RN*, *IL20RB*, *TLX3*, and *ZEB2* (Table S6). The top five genes with hypermethylated DMRs in the FUN tissue were annotated to the *MIR9-1*, *HOXC11*, *FKBP5*, *MIR99A*, and *ZNF574* genes.

To gain a deeper understanding of the biological functions associated with infection-specific DMRs, all genes annotated from these DMRs were tested for gene enrichment and pathway analysis using PANTHER (FDR < 0.05) and IPA. In the dLN, FUN, and PYL tissues, a total of 214, 206, and 48 genes, respectively, were used for the functional analysis.

The GO terms overrepresented by dLN and FUN DMRs were mainly related to the regulation of dephosphorylation, macromolecule biosynthetic process, gene expression/transcription activity, cellular metabolic process, and DNA binding (Table S8). PYL DMRs showed overrepresented GO terms associated with the pattern specification process, regionalization, regulation of transcription/gene expression, macromolecule biosynthetic process, and DNA binding (Table S8).

In IPA analysis, dLN, FUN, and PYL tissues exhibited nine, nine, and two significant networks (score > 10), respectively (Table S9). dLN networks were mainly related to gene expression, the cell cycle, cancer, and cell signaling. Networks in the FUN tissue were related to the cell cycle, cancer, and cell development. Additionally, IPA-enriched canonical pathways revealed important functional networks, including RAR activation (FUN, PYL, and dLN), WNT/β-catenin signaling (dLN), the transcriptional regulation of pluripotent stem cells (FUN and dLN), and NGF-stimulated transcription (FUN and PYL) (Table S10).

**Figure 5:**
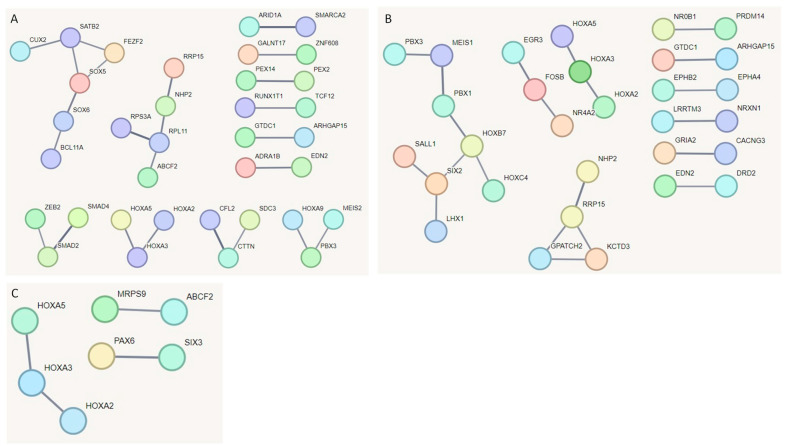
STRING network analysis of DMR genes from (**A**) dLN, (**B**) FUN, and (**C**) PYL tissues. Each node represents a gene, and the lines represent predicted interactions (with a minimum confidence score of 0.7). The line thickness indicates the strength of the supporting data for the predicted interactions.

Furthermore, we constructed functional protein association networks for dLN, FUN, and PYL tissues using genes from the DMRs (Figure 5). In dLN, DMRs showed two interaction hubs with > three genes per hub, four interactions with three genes, and six interactions with only two genes (Figure 5). In the FUN tissue, we observed one interaction with eight genes, one with four genes, and several interactions that had fewer than four genes. In the PYL tissue, we observed only three interactions. *HOX2*, *HOX3*, and *HOX5* genes were observed in all tissues. Genes related to immune response such as *BCL11A*, *EGR3*, *FOSB*, *SATB2*, *SMAD2*, *SMAD4*, and *ZEB2* were part of the interactions.

### 2.6. Functional Analysis of DMGs and DPMGs

We merged all DMGs with the DPMGs to explore the biological functions associated with the DMRs exclusively located on genes and within promoter regions (−10 kb to +1 kb from the TSS). We conducted several assessments, including gene enrichment, pathway, and network analyses. From the dLN, we identified 94 genes (83 DMGs and 30 DPMGs), and from the FUN, we obtained 74 genes (55 DMGs and 25 DPMGs), resulting in a significant overrepresentation of GO terms, IPA networks, and enriched canonical pathways. However, no significant results were obtained for PYL and DUO DMGs and DPMGs.

The DMGs and DPMGs in both FUN and dLM revealed GO terms predominantly associated with the regulation of transcription and expression, cellular metabolic processes, and macromolecule biosynthetic processes (Table S11). In the FUN tissue, five IPA networks were identified related to organismal injury and abnormalities, nervous system development and function, and immunological disease/inflammatory disease/inflammatory response network with eight focus molecules (Table S12). In dLN, six IPA networks were identified related to cellular development and proliferation, organ morphology, cancer, immunological disease, and inflammatory response (Table S12). Additionally, IPA-enriched canonical networks revealed important functional pathways, including RAR activation (FUN and dLN), IL-15 production (FUN), the activation of anterior HOX genes in the hindbrain during early embryogenesis (FUN and dLN), NGF-stimulated transcription (FUN), and RNA Polymerase III transcription (dLN) (Table S13).

**Figure 6:**
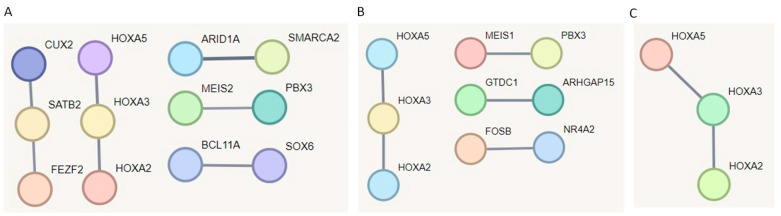
STRING network analysis of DMR and DPMG genes from (**A**) dLN, (**B**) FUN, and (**C**) PYL tissues. Each node represents a gene, and the lines represent predicted interactions (with a minimum confidence score of 0.7). The line thickness indicates the strength of the supporting data for the predicted interactions.

In addition, we constructed functional protein association networks for dLN, FUN, and PYL tissues using the combined list of DMGs and DPMGs. We observed limited interactions in both tissues (Figure 6). In dLN, DMGs, and DPMGs showed two interaction hubs with three genes per hub and three interactions with only two genes. In the FUN, we observed only four interactions, each involving a few genes. In the PYL, we identified a single interaction involving three genes (Figure 6). *HOX2*, *HOX3*, and *HOX5* genes were consistently observed in all analyzed tissues.

### 2.7. Effect of Differential Methylation on Gene Expression

DNA methylation has the potential to modulate gene expression to varying degrees. Therefore, we overlayed the DMPs and DMRs entirely located on genes or those on TSS (−10 kb to +1 kb from the TSS) with DEGs (FDR < 0.05 and |log2FC| > 1.5) in cattle infected with *O. ostertagi* (Under review). We utilized 648 DEGs in FUN and 522 DEGs in dLN for the expression data.

For the methylation DMP data, we utilized 177 genes that contained the FUN DMPs, 321 genes that contained dLN DMPs, 69 genes with FUN DMPs located on their TSS regions/promoters, and 163 genes with dLN DMPs located on the TSS regions/promoters. For the methylation DMR data, we utilized 51 genes that entirely contained FUN DMRs, 67 genes that contained dLN DMRs, 15 genes that contained PYL DMRs, 24 genes from FUN DMRs located on TSS regions/promoters, 28 genes from dLN DMRs located on TSS regions/promoters, and seven genes from PYL DMRs located on TSS regions/promoters.

A total of 10 DMPs and 3 DMRs in dLN, and 6 DMPs in the FUN tissue exhibited a correlation with expression patterns involving 11 genes (Table 1). Among these 11 genes, three are related to cancer (*SLC12A5*, *SORCS1*, and *TP63*), and six are immune-related genes, including *AHR*, *LRFN5*, *NTRK2*, *RSPO2*, *SAMSN1*, and *TFEC*. Notably, two hypermethylated DMPs were located in promoter regions in the dLN with an expected repression of gene expression (Table 1). In addition, we examined TFs and TF cofactors and identified five genes as TFs.

### 2.8. Gene-Enriched Analysis of Methylation Array Cattle Genes

Analysis was performed on a total of 5556 cattle genes from the mammalian methylation array to assess GO enrichment. The results indicated significant enrichment primarily associated with development, growth, transcriptional regulation, and metabolism (Table S14).

## 3. Discussion

In this study, we characterized the genome-wide methylation profiles of four different cattle tissues from *O. ostertagi*-infected and non-infected animals using a custom-designed mammalian methylation array [6]. We tested the DNA methylation in 31,246 cattle CpG sites using the Infinium array “HorvathMammalMethyl-Chip40” in 37 samples [6]. After removing low-quality CpG sites, we successfully analyzed 31,107 cattle CpGs associated with a total of 5082 cattle genes.

The mammalian methylation array showed high coverage of conserved cytosines and demonstrated high fidelity in humans, rats, and mice [6]. Notably, we have recently utilized this array to investigate DNA methylation patterns in relation to feed efficiency traits in dairy cattle [10]. The selection of CpG probes in this array was based on highly conserved sequences [6]. Gene enrichment analysis revealed that the genes represented in this array were associated with development, growth, transcriptional regulation, metabolism, cancer, mortality, aging, and survival [6]. Subsequent enrichment analyses performed on all cattle genes represented in the array displayed similar outcomes, mainly highlighting pathways associated with development, growth, transcriptional regulation, and metabolism.

In cattle, methylation studies have been performed focusing on various traits, including diseases [12,13,14], aging [15], reproduction [16,17,18], or embryonic development [19,20]. However, most of them have limited coverage or a limited number of samples, and some of them utilized bisulfite sequencing. Parasite infections are known to modulate the host transcriptome and epigenome by downregulating immune responses and promoting cell proliferation [23]. A recent study assessed the impact of cattle parasite infection on DNAm content; the findings suggest a potential relationship between parasitic resistance and global DNAm [24]. However, this study measured the relative levels of methylated DNA using an ELISA-based method [24], which is not suitable for a precise DNAm estimation and which is limited to the detection of large changes in global DNAm [5].

To our knowledge, this study marks the first attempt to characterize genome-wide methylation profiles in cattle infected with a GI nematode using a custom DNAm array. Methylation arrays, widely employed in human studies, are particularly valued for their ability to gather information from individual loci reliably and cost-effectively [5].

When comparing infected with uninfected animals, we identified infection-specific DMPs with 5% Δβ in two tissues—dLN and FUN. The dLN exhibited a higher number of DMPs, with both tissues displaying more hypomethylated DMPs. Around 12% of these DMPs were shared between dLN and FUN, suggesting that differential epigenetic regulations exist in these tissues during nematode infections. However, these shared DMPs, involving 263 genes, encompassed multiple genes associated with the immune response, such as *BCL11A*, *BMP4*, *DAPL1*, *IFNG*, *IL20RB*, *IKZF1*, *LRFN5*, *LYL1*, *LZTS1*, *MAP3K7*, *MYB*, *NFATC3*, *NTRK2*, *ONECUT1*, *PRDM1*, *PROX1*, *PSMD7*, *ROBO1*, *RRAGD*, *SATB1*, *SMAD7*, *TFEC*, *TLE1*, *TLE3*, *TP63*, *TPRG1*, *TXNL1*, *ZEB2*, and *ZBTB7B*.

For example, a DMP located in the promoter region of the *IFN-γ* (or *IFNG*) gene, crucial in immunity against some pathogens [28], was identified in both FUN and dLN. The gene *LRFN5* plays important roles in host immune response, including the negative regulation of inflammation and macrophage activation [29]. In a recent study with goats, the gene *LRFN5* was found to be associated with immune response traits in the indigenous ecotypes [30]. A copy number variation was also identified in the *LRFN5* gene in indigenous Nguni cattle, which are resistant to diseases and parasite infections [31]. Notably, *LRFN5* showed two DMPs in dLN—one hypermethylated in the promoter region and one hypomethylated in the intron. In FUN tissue, *LRFN5* exhibited a hypomethylated DMP within its intronic region. SATB1 is a transcription factor and an important chromatin organizer, and it plays an essential role in the immune system [32,33]. In FUN, one hypomethylated DMP was identified in the exon region of *SATB1*. Interestingly, in dLN, 28 DMPs (27 hypermethylated) were identified, with 21 being entirely located on its gene bodies. Also, one of these 28 dLN DMPs was among the top five hypermethylated DMPs in its intronic region of the *SATB1*.

In humans, the TFEC is a macrophage-specific transcription factor that plays a role in regulating *IL4* expression [34]. In dLN, four hypomethylated DMPs were identified in the *TFEC* gene, including two in intronic regions and two in distal intergenic regions. In FUN, two hypomethylated DMPs were identified in the distal intergenic regions of the *TFEC* gene. The TF *ZEB2* is involved in the epithelial to mesenchymal transition and plays a role in the immune system through expression in several immune cells, including macrophages, monocytes, and B, T, and NK cells [35]. In dLN, we detected 29 hypo/hypermethylated DMPs, with 22 of them located in intron and promoter regions of the *ZEB2* gene. In FUN, three hypermethylated DMPs were observed in intronic regions of this gene.

Our analysis of the infection-specific DMPs revealed enriched GO terms related to DNA binding and transcription regulation, possibly influencing gene expression. Furthermore, IPA analysis highlighted important immune-related enriched canonical pathways for WNT/β-catenin signaling, CDX GI cancer signaling, chronic myeloid leukemia signaling, IL-12 signaling and production in macrophages, Th1 and Th2 activation, IL-7 signaling, and IL-4 and -13 signaling in dLN and/or FUN tissues. Overall, these findings suggest that some of the infection-specific DMPs identified in this study are functionally associated with immune responses and defense against *O. ostertagi*.

The Wnt/β-catenin pathway is essential to embryonic development, cell proliferation, differentiation, migration, and apoptosis, and it is linked to immunosuppressive function in tumors [36]. During *Trichinella* spp. infection, there is an elevated expression of genes involved in downregulating the canonical Wnt/β-catenin signaling in nurse cells, suggesting a need to suppress this pathway for the parasite’s survival [37]. Furthermore, in a study using the murine helminth models, it was shown that the gene deletion for SETD7, a histone lysine N-methyltransferase mediating methylation of target genes, has rendered mice resistant to infection by *Trichuris muris* [38]. This study also shows that SETD7 controls intestinal epithelial cell turnover by modulating multiple signaling pathways, which include the Wnt/β-catenin cascade. Obviously, further studies are warranted to elucidate the exact role of an enriched Wnt/β-catenin signaling pathway, as shown in the present study. In general, T helper type 2 (Th2) immune responses are key to protection against nematode parasites [39]. In the mouse model, helminth infections typically upregulate Th2 but concomitantly downregulate Th1 responses. Previous studies in rodent models have shown that IL-4 and IL-13, primarily produced by Th2 cells and basophils, significantly contribute to protective immunity against GI nematodes, while IL-12 and IFN-γ, which both are key Th1 cytokines, can inhibit this protective immunity [40]. The activation of both Th1 and Th2 responses via *O. ostertagi* infection is intriguing. Future studies will focus on the role of Th1 responses elicited via the infection, which may be speculated as a feature of the bovine immunity to the nematodes or represent a mechanism of immune evasion. 

In addition, DMPs that were entirely located on genes or TSS (−10 kb to +1 kb from the TSS) could potentially influence gene expression and transcription. Several DMPs were identified on genes associated with immune functions, including *CLEC11A* (dLN), *EPHB2* (FUN), *FKBP5* (dLN and FUN), *FOS* (FUN), *FOSB* (dLN), *FOXP1* (dLN and FUN), *IL17B* (FUN), *IRF1/5* (dLN), *LEF1* (dLN), *LOC534155* (dLN), *LRIG3* (dLN), *NTRK3* (FUN), *PLPP4* (dLN), *PSMD14* (FUN), *RARA* (dLN), *RSPO2* (dLN), *SATB2* dLN), *SMAD4* (dLN), *SMAD6* (FUN), *TCF4/12* (dLN), *TGFB3* (dLN), *TLE4* (dLN), *TMEM88* (dLN), and *TRAF3* (dLN).

For instance, the *EPHB2* gene plays a role in monocyte adhesion and transmigration [41], and its upregulation has been observed during post-malaria infection in mice, modulating inflammatory responses [42]. One hypermethylated DMP was identified in the promoter region of this gene in FUN. Although its functions are not well understood, IL17B plays essential roles in host defense and inflammation [43]. IL17B has been associated with tumor progression [44] and is increased during intestinal inflammation [45]. Two hypomethylated DMPs were identified in the exonic regions of this gene in FUN. *LEF1* (lymphoid enhancer binding factor 1) is highly expressed in dLN and is involved in the Wnt signaling pathway, and it plays roles in B-cell proliferation and as T-cell receptor enhancer [46]. Three hypermethylated DMPs were identified in the *LEF1* gene’s intronic regions in dLN. In a previous study of cattle, the *LEF1* gene was identified as a DEG in bta-miR-192 mutant cells exposed to *Escherichia coli* [47]. Additionally, the *TMEM88* gene, also related to the Wnt pathway, showed DMPs in dLN [48].

SATB2, highly expressed in GI tissue, is associated with colorectal cancer and the regulation of GI inflammation [49]. One hypomethylated DMP was identified in the intronic region of this gene in dLN. SMAD proteins act as tumor suppressors and modulate host signaling during bacterial and viral infections [50]. Moreover, the production of IgA, an abundant mucosal antibody, is modulated through the SMAD pathway, maintaining the mucosal defensive barrier [51]. A previous study of mice identified an effect of *Toxoplasma gondii* in the suppression of the SMAD2/3/4 pathway [52]. SMAD4 plays essential roles in promoting T-cell function and mediating TGF-β signaling in T-cells [53]. Two hypomethylated DMPs were found in the *SMAD4* gene in dLN, while two DMPs were identified in the *SMAD6* gene in FUN.

TLE proteins play essential roles in immune cells (macrophages and lymphocytes), and their levels impact cancer treatment outcomes and drug efficacy [54]. TLE4 is known to modulate the epigenetic silencing of *IFN-γ* expression [55]. Notably, two hypomethylated DMPs were found on the *TLE4* gene in dLN. These DMPs, located on genes and promoter regions related to immune functions, might act as potential epigenetic drivers in nematode infections.

The analysis of infection-specific DMRs with a Δβ of 10% revealed more DMRs in dLN and FUN tissues, including more hypomethylation (Δβ ≤ −10%). These DMRs were associated with several immune-related genes across all tissues tested, including *C8B* (FUN), *BCL11A* (dLN and FUN), *EPHB2* (FUN), *EGR3* (FUN), *FKBP5* (FUN dLN), *FOSB* (FUN), *FOXP1* (FUN and dLN), *IL17B* (FUN), *IL1RN* (FUN), *IL20RB* (FUN), *IGBP1* (dLN), *IRX5* (dLN), *NTRK3* (FUN), *OIT3* (dLN), *RAB2A* (dLN), *RARA* (dLN), *SATB1/2* (dLN), *SMAD2* (dLN and DUO), *SMAD4* (dLN), *TLE1/4* (dLN), *TLX3* (FUN), and *ZEB2* (all four tissues).

These genes are important for host immune responses, and some of them were already identified in the DMP analysis (e.g., *EPHB2*, *FKBP5*, *IL17B*, *NTRK3*, *RARA*, *SATB1*, *SATB2*, *SMAD4*, *TLEs*, and *ZEB2*). The early growth response genes encompass four members of transcription factors: EGR1, EGR2, EGR3, and EGR4 [56]. EGR2 and EGR3 play a pivotal role in regulating the immune system via the activation of B- and T-cells [56]. *FKBP5* is an anti-influenza host factor [57], and it plays several roles in immunoregulation [58]. In an earlier cattle QTL mapping study, the most significant associations with bovine tuberculosis were located on the *FKBP5* gene [59]. In FUN, one of the top five hypermethylated DMRs was found in the intron region of the *FKBP5* gene. In dLN, one of the top 10 hypermethylated DMR was also annotated on the intron region of the *FKBP5* gene. The *Fos* gene family has four members (*FOS*, *FOSB*, *FOSL1*, and *FOSL2*) that contribute to cell proliferation, differentiation, and immunomodulation [60,61]. The *OIT3* gene in humans is mainly expressed in the liver, small intestine, and duodenum, and its functions are related to the metabolism of macrophages and cancer progression [62]. As mentioned before, SMAD proteins are important mediators during infection [50,51,52]. In dLN, one of the top five hypermethylated DMRs was annotated to the distal intergenic region of the *SMAD2* gene. In addition, the *RARA* gene plays important roles associated with immune activation [63]. Three retinoic acid receptors (RARs), namely RAR-α, RAR-β, and RAR-γ, are encoded by the *RARA*, *RARB*, and *RARG* genes, respectively. In dLN, the top hypermethylated DMR was annotated to the intronic region of the *RARA* gene. Interestingly, retinoic acid, a metabolite of vitamin A and high-affinity ligands for RARs, modulates allergic airway disorders by inhibiting Th2/Th17 response and enhancing Treg cells [64]. Further, treatment with the retinoic acid receptor (RARα/β) agonist Am80 enhances IL-6-dependent mucosal inflammation and exacerbates *T. muris*-induced inflammation [65]. Given the complexity of the RAR-mediated biological network, its role in host immunity to nematode infection warrants in-depth investigation. 

To deepen our understanding of the biological functions linked to infection-specific DMRs, we subjected all genes annotated from these DMRs to overrepresentation, IPA pathway, and network analyses. Overrepresented tests across all tissues showed similar terms, including the regulation of transcription, regulation of RNA biosynthetic process, regulation of gene expression, and macromolecule biosynthetic process. This result suggests that the identified DMRs may affect gene expression. IPA-enriched canonical pathways revealed crucial functional pathways, including pathways related to immune functions, such as RAR activation (all tissues), WNT/β-catenin signaling (dLN), and NGF-stimulated transcription (FUN and PYL). The signaling pathway of RAR includes genes like *RARA*, *HOX3/5*, *DHRS3*, and others, which significantly influence immune responses like protective immunity, immune homeostasis, and lymph node organogenesis [63]. Nerve growth factor (NGF) can modulate both neuronal and immune functions, and upregulated *NGF* expression is associated with inflammatory diseases [66].

Functional protein association networks revealed networks with interactions related to immune genes in FUN and dLN; these genes include *BCL11A*, *EGR3*, *FEZF2*, *FOSB*, *NR4A2*, *SATB2*, *SMAD2*, *SMAD4*, and *ZEB2*. *BCL11A* gene encodes a TF with functions related to lymphopoiesis [67]. The *FEZF2* gene is related to immune tolerance in mice [68]. *NR4A2* was associated with inflammatory and metabolic functions [69]. In addition, the *HOX2*, *HOX3*, and *HOX5* genes were observed in interactions present in all tissues. *Homeobox* (*HOX*) are highly conserved genes with several important functions related to development, cell differentiation, apoptosis, and cancer [70].

Similarly, we verified the biological functions linked to the DMGs and DPMGs. Similar to DMR results, the DMG/DPMG overrepresentation test in FUN and dLN showed GO terms mainly related to the regulation of RNA and gene expression, and macromolecule biosynthetic and metabolic processes, indicating that the differentially methylated genes may affect gene expression. The IPA analysis revealed networks related to the inflammatory response in both FUN and dLN, suggesting an effect of methylation elicited via *O. ostertagi* infection. The IPA canonical pathways also showed pathways relating to immune functions including RAR activation, IL-15 production, and NGF-stimulated transcription. In mice, the eggs of *Schistosoma mansoni* can cause granulomas and elevate levels of NGF in the central nervous system and liver [71,72]. The increase in NGF-stimulated transcription shown in this study may reflect a direct or indirect effect of *O. ostertagi* on the induction of NGF and, consequently, NGF-induced gene transcription. 

As mentioned before, RAR and NGF can influence immune functions [63,66], and IL-15 plays major roles in immune and inflammatory responses [73]. The IL-15 production-enriched canonical pathway in FUN had three molecules, including EPHB2, FGFR2, and NTRK3. In humans, the *EPHB2* gene is highly expressed in the GI tract and is related to inflammatory responses [41,42]. *NTRK3* expression was associated with immune infiltration in humans [74].

We further integrated our methylation results with RNA-seq data [75], and we identified 11 genes, including six related to immune functions (*AHR*, *LRFN5*, *NTRK2*, *RSPO2*, *SAMSN1*, and *TFEC*), and three related to cancer (*SLC12A5*, *SORCS1*, and *TP63*). In addition, five of the 11 genes were classified as TFs (*AHR*, *EBF2*, *PRDM6*, *TFEC*, and *TP63*). AHR responds to microorganisms, toxins, metabolism, or diet, and it has essential functions in immune responses [76]. The *AHR* gene protects the GI against inflammation and prevents intestinal infections [77]. A study with mice infected with *Trypanosoma cruzi* showed that AHR can affect parasite replication and infection [78]. Another study showed that the treatment with AhR ligand can result in beneficial immune effects during leishmaniasis [79].

## 4. Material and Methods

### 4.1. Experimental Design and Tissue Collection

The Holstein steers used in this study were from the Beltsville Agricultural Research Center (BARC) Dairy Unit and were raised helminth-free from birth. These animals were weaned at three months of age and had free access to water and feed. All calves were fed with milk replacer until 3 months of age and then transitioned to 18% Calf Growers and hay; starting from ~6 months of age, all animals were finished with feedlot pellet and hay (Farmers Cooperative Association, Inc., Frederick, MD, USA). Trace minerals were supplemented using the Trace Mineral Livestock Blocks. The propagation of *O. ostertagi* using helminth-free calves was conducted as described previously [6]. All 10 animals in this study either were subjected to an oral trickle infection with 1000 *O. ostertagi* L3 per day, 5 days per week, for four weeks, or received tap water as control, at the age of approximately 11 months. Animals were then euthanized on day 30 post-infection (dpi). During infection, animals were monitored for body weight changes and fecal egg counts. At necropsy, samples of the duodenum (DUO) mucosa, abomasal fundic (FUN), pyloric (PYL) mucosa, and draining lymph nodes (dLN) were collected, snap-frozen in liquid nitrogen, and stored at −80 °C until processed for genomic DNA and total RNA isolation. Postmortem abomasal content was collected for worm load and pH determination, and total dLN was collected for total dLN weight measurement. Due to limitations in the number of animals that could be euthanized per day, the infected animals were euthanized 2 days apart at 29 or 31 dpi. Therefore, for convenience, all five animals in the infected group were collectively named 30 dpi, and all five uninfected animals were named 0 dpi. Out of the 40 samples collected, 37 (9 dLN, 9 FUN, 9 PYL, and 10 DUO) were utilized in this study. Three samples, one FUN from the infected group, one PYL from the control group, and one dLN from the control group, were excluded due to low-quality data. The animal care and use protocol was approved by BARC IACUC (protocol number 16-019).

### 4.2. DNA Methylation Array Information

DNA methylation data from the four tissues were generated using the custom Infinium array “HorvathMammalMethyl-Chip40”, which is also known as the mammalian methylation array [6]. This array measures 37,492 CpGs across over 200 species, and it focuses on highly conserved CpGs [6]. The chip manifest file of the mammalian methylation array can be found at the Gene Expression Omnibus (GEO) at NCBI as platform GPL28271.

### 4.3. Probe Normalization

The SeSaMe package was utilized to normalize the data and obtain the beta values (β-values) for each probe [26]. β-values represent the methylation levels of the probes between 0 (completely unmethylated) and 1 (fully methylated). The formula employed is β = M/(M + U + 100), where M represents intensity for methylation, and U stands for intensity for unmethylation.

### 4.4. Probe Mapping and Annotation

Probe sequences were mapped using the package QuasR (version 1.35.1) [80] with parameters -k 2-strata-best -v 3 and bisulfite = “undir” to align the enlarged set of probe sequences. From the initial 37,492 CpGs in 200 species, a total of 31,252 CpGs was mapped to the cattle reference genome (ARS-UCD1.2) [25]. Six probes located on unknown chromosomes were removed, resulting in a final count of 31,246 CpGs. Genome coordinates for each CpG in cattle were obtained from the GitHub page of the Mammalian Methylation Consortium (https://github.com/shorvath/MammalianMethylationConsortium/tree/v1.0.0 (accessed on 15 August 2022)). Following the alignment, the CpGs were annotated to genes based on the distance to the closest TSS (transcription start site) using the Chipseeker package (version 1.33.1) [81]. CpGs were categorized by their genomic locations such as exon, intron, 3′ UTR, 5 UTR, promoter region (−10 kb to +1 kb from the nearest TSS), downstream, or intergenic region.

### 4.5. Quality Control

Quality control was performed with all 37 samples and 31,246 CpGs. We obtained the distribution of the β-values and M-values for each sample using the minfi R package (version 1.18.4) [82]. The β-values for each sample and tissue were converted to M-values (M = log_2_ [M/U]) using the beta2m function from the wateRmelon R package (version 1.16.0) [83]. Although the β-value is more biologically interpretable, the M-value is necessary for conducting differential methylation analysis [84]. We also generated the principal component analysis (PCA) of the 37 samples based on the β-values of each sample for the 31,246 CpGs using the *gplots* package from R (version 4.2.1) to assess sample quality and identify potential outliers and batch effects.

### 4.6. Probe Filtration at SNP Sites

Two different strategies were utilized to identify the potential interference of SNPs on CpG probes among the 31,246 cattle CpGs. First, we compared our 31,246 cattle CpGs against the location of 67,965,046 SNPs identified in 1168 Holsteins with MAF > 5% using the BEDtools (version 2.30.0) [85] intersect option. Secondly, we utilized the MethylToSNP package (version 0.99.0) [27] to identify probes affected by adjacent SNPs. MethylToSNP leverages methylation patterns observed at SNP sites to predict the presence of SNPs [27].

### 4.7. Differentially Methylated Positions (DMPs)

DMPs were identified using the dmpFinder function from the *minfi* R package (version 1.18.4) [82] based on the M-values at FDR < 0.05 and |Δβ| ≥ 5%. The methylation level of each CpG probe is denoted as a β value. The Δβ of each CpG site represents the difference in average β values between the infected animals and the uninfected controls. A CpG site with |Δβ| ≥ 5% and FDR < 0.05 was considered a DMP or a differentially methylated CpG site. Specifically, a CpG site was considered hypermethylated if Δβ ≥ 0.05 or hypomethylated when Δβ ≤ −0.05, representing a 5% difference in methylation levels.

### 4.8. Differentially Methylated Regions (DMRs)

DMRs were identified in each tissue using the Bumphunter function from the *minfi* package (version 1.46.0) [86]. The identification criteria included a maximum allowable gap of 250 bp between probe start positions for probes to be grouped into the same region, a requirement of a minimum of three probes per region, a cut-off value of 0.1 (which corresponds to a 10% difference in the methylation levels), and a significance threshold of *p*-value ≤ 0.001.

### 4.9. DMP and DMR Annotation

The significant DMPs and DMRs for each tissue were annotated based on the distance to the closest transcriptional start site (−10 kb to +1 kb from the nearest TSS) using the ChIPseeker package (version 1.36) [81].

### 4.10. Differentially Methylated Genes (DMGs) and Differentially Promoter-Methylated Genes (DPMGs)

We further investigated genes that either themselves or whose TSS regions (−10 kb to +1 kb from the TSS) entirely contained the DMRs. We classified them as either differentially methylated genes (DMGs) or differentially promoter-methylated genes (DPMGs), respectively. From these, we compiled a gene list encompassing all DMGs combined with the DPMGs for the downstream analysis.

### 4.11. Statistical Overrepresentation Test

We conducted a statistical overrepresentation analysis using PANTHER (version 18.0) [87] with the PANTHER GO-slim datasets for Biological Process (BP), Molecular Function (MF), and Cellular Component (CC) from the DMPs and DMRs. This analysis was conducted using Fisher’s exact test, with an adjustment for a false discovery rate of FDR < 0.05. This test was conducted for each functional category to determine whether there was any significant overrepresentation of any genes on the test list (genes annotated from the DMPs, DMRs, DMGs, and DPMGs) in comparison to the cattle reference list. To mitigate bias stemming from the design of the mammalian methylation array, which predominantly focuses on well-conserved sequences among mammals [6], we utilized only 5082 cattle genes from the array as the background reference list, instead of all cattle genes.

### 4.12. IPA Pathways

We utilized QIAGEN Ingenuity Pathway Analysis (IPA) (version 94302991) [88] to explore signaling and metabolic pathways for genes with relevant biological functions from the gene list obtained from the DMRs, DMGs, and DPMGs. The “Core Analysis” function was performed with default parameters, including networks with a maximum of 35 molecules, only molecules with experimentally observed confidence, and human species only. Networks with scores ≥ 10 were deemed significant. The enriched canonical pathways were identified based on criteria, applying a threshold of −log (*p*-value) > 2 or a *p*-value of < 0.01. Additionally, z scores ≥ 2 indicated predictions of activation, while z scores ≤ −2 suggested predictions of inhibition.

### 4.13. Protein–Protein Interaction Networks

We constructed functional protein association networks using STRING (version 12.0) [89]. STRING utilizes different sources of information, such as text mining, experiments, databases, coexpression, neighborhoods, gene fusion, and cooccurrence. We analyzed the gene list from the DMRs, DMGs, and DPMGs of each tissue to generate the networks using a high-confidence-level mode (0.70), including all aforementioned sources of interactions.

### 4.14. Methylation Integration with RNA-Seq Data

We compared our DNA methylation results with a previous study that identified differentially expressed genes (DEGs) associated with cattle infected with *O. ostertagi* [75]. All DEGs were compared with the DMGs and DPMGs of which either themselves or their TSS regions (−10 kb to +1 kb from the TSS) entirely contained the DMRs. Any genes overlapping in both methylation and RNA-seq data were further checked for transcription factors (TF) using the AnimalTFDB for cattle (version 4.0), which includes 1445 TFs and 939 TF cofactors [90].

### 4.15. Gene-Enriched Analysis of Array Cattle Genes

A total of 5094 cattle genes derived from the mammalian methylation array, corresponding to 31,252 cattle CpGs, were further analyzed for GO enrichment (BP) using GREAT (version 4.0.4) [91] with default settings. The analysis was performed on the human hg38 genome, employing a hypergeometric FDR < 0.05. To facilitate this, cattle coordinates were converted to human (hg38) using UCSC LiftOver with default settings [92].

## 5. Conclusions

The *O. ostertagi*-induced, differentially methylated positions and regions and the resultant functional outcomes demonstrated in this study indicate that nematode infections can play an essential role in significantly influencing host gene methylation responses, resulting in the modulation of gene expression. As expected, immune-related genes and pathways in the draining lymph nodes of the abomasum (dLN) and abomasal fundus (FUN) where the parasite resides are heavily affected during the infection. The infection-induced host response is highly complex; thus, the global host immune responses to the infection are also mixed. In addition, gene methylation may only be one of the mechanisms the host responds to and uses to shape the immunity to infection. The pathways including WNT/β-catenin signaling, Th1 and Th2 activation, RAR activation, NGF-stimulated transcription, IL-15 production, and IL-4, 7, 12, and 13 signaling are the top pathways that the nematode infection elicits, which could lead to eventual protection or immune evasion. Some of the differentially methylated target genes associated with host immune responses may be further investigated to demonstrate host resistance and gene products that can be used as vaccine candidates. Integration with RNA-seq data additionally revealed genes with immune roles and cancer, some of which were TFs. These findings enhance our comprehension of the epigenetic regulatory framework in nematode-infected cattle, spotlighting potential areas of further investigation.

## Figures and Tables

**Table 1:** Genes obtained from integrative analysis of differential methylation and RNA-seq data.

Gene	Gene Name	Tissue	Type	CpG	Chr	Start	End	Annotation	Mean Δβ	log2FC	Direction	TF Family
*TP63*	tumor protein p63	dLNs	DMP located on gene	cg02197333	1	77479563	77479564	Exon (exon 4 of 14)	−0.068	2.08	inconsistent	P53
*TFEC*	transcription factor EC	dLNs	DMP located on gene	cg06760134	4	52303479	52303480	Intron (intron 1 of 7)	−0.095	4.08	inconsistent	bHLH
*TFEC*	transcription factor EC	dLNs	DMP located on gene	cg03543715	4	52303494	52303495	Intron (intron 1 of 7)	−0.120	4.08	inconsistent	bHLH
*PRDM6*	PR/SET domain 6	dLNs	DMP located on gene	cg13468491	7	30708268	30708269	Intron (intron 3 of 7)	−0.101	−2.21	consistent	zf-C2H2
*NTRK2*	neurotrophic receptor tyrosine kinase 2	dLNs	DMP located on gene	cg19217250	8	78023690	78023691	Exon (exon 8 of 12)	−0.180	−1.60	consistent	-
*RSPO2*	R-spondin 2	dLNs	DMP located on gene	cg06896863	14	56498391	56498392	Exon (exon 3 of 5)	−0.106	−3.23	consistent	-
*LRFN5*	leucine rich repeat and fibronectin type III domain containing 5	dLNs	DMP located on gene	cg15469181	21	51632828	51632829	Intron (intron 3 of 3)	−0.108	−3.96	consistent	-
*LRFN5*	leucine rich repeat and fibronectin type III domain containing 5	dLNs	DMP located on TSS	cg04784672	21	51329097	51329098	Promoter (<=1kb)	0.057	−3.96	consistent	-
*SORCS1*	sortilin related VPS10 domain containing receptor 1	dLNs	DMP located on gene	cg21229793	26	27556508	27556509	Intron (intron 26 of 26)	−0.088	−1.74	consistent	-
*SORCS1*	sortilin related VPS10 domain containing receptor 1	dLNs	DMP located on TSS	cg15043841	26	28128919	28128920	Promoter (<=1kb)	0.052	−1.74	consistent	-
*SAMSN1*	SAM domain, SH3 domain and nuclear localization signals 1	FUN	DMP located on gene	cg16665024	1	22629640	22629641	Intron (intron 1 of 7)	−0.053	1.83	inconsistent	
*EBF2*	EBF transcription factor 2	FUN	DMP located on gene	cg05217279	8	73218285	73218286	Intron (intron 6 of 15)	−0.070	2.51	inconsistent	COE
*SLC12A5*	solute carrier family 12 member 5	FUN	DMP located on gene	cg17424512	13	74775035	74775036	Exon (exon 6 of 26)	−0.060	−1.63	consistent	-
*SLC12A5*	solute carrier family 12 member 5	FUN	DMP located on gene	cg09355828	13	74778288	74778289	Exon (exon 8 of 26)	−0.084	−1.63	consistent	-
*LRFN5*	leucine rich repeat and fibronectin type III domain containing 5	FUN	DMP located on gene	cg08683365	21	51635204	51635205	Intron (intron 3 of 3)	−0.097	3.80	inconsistent	-
*LRFN5*	leucine rich repeat and fibronectin type III domain containing 5	FUN	DMP located on gene	cg15469181	21	51632828	51632829	Intron (intron 3 of 3)	−0.126	3.80	inconsistent	-
*TFEC*	transcription factor EC	dLNs	DMR located on gene	-	4	52303479	52303494	Intron (intron 1 of 7)	−0.825	4.08	inconsistent	bHLH
*AHR*	aryl hydrocarbon receptor	dLNs	DMR located on gene	-	4	25820158	25820214	Intron (intron 2 of 10)	0.283	1.91	consistent	bHLH
*PRDM6*	PR/SET domain 6	dLNs	DMR located on gene	-	7	30708244	30708355	Intron (intron 3 of 7)	−0.427	−2.21	consistent	zf-C2H2

## Data Availability

The RNA-seq data were deposited in the NCBI Sequence Read Archive (SRA) under the accession number PRJNA1030293.

## Supplementary Materials

The following supporting information can be downloaded at https://www.mdpi.com/article/10.3390/ijms26010089/s1.

## Abbreviations

BARC	Beltsville Agricultural Research Center
BPs	biological processes
CC	cellular component
DEG	differentially expressed gene
DMGs	differentially methylated genes
DMPs	differentially methylated positions
DMRs	differentially methylated regions
DNAm	DNA methylation
DPI	day post-infection
DPMGs	differentially promoter-methylated genes
GEO	Gene Expression Omnibus
GI	Gastrointestinal
HOX	Homeobox
IPA	ingenuity pathway analysis
LEF1	lymphoid enhancer binding factor 1
MeDIP	methylated DNA immunoprecipitation
MF	molecular function
MSP	methylation-specific PCR
NGF	nerve growth factor
PCA	principal component analysis
RAR	retinoic acid receptor
RNA-seq	RNA sequencing
TF	transcription factor
Th2	T helper type 2
TSS	transcription start site
β-values	beta values
